# Comparison of clinical characteristics and outcomes between patients with complicated pleural infection caused by *Streptococcus anginosus* group and *Klebsiella pneumoniae*

**DOI:** 10.1515/pp-2025-0017

**Published:** 2025-09-26

**Authors:** Chang Ho Kim, Ji Eun Park, Sun Ha Choi, Yong Hoon Lee, Hyewon Seo, Seung Soo Yoo, Shin Yup Lee, Seung Ick Cha, Jaehee Lee

**Affiliations:** Department of Internal Medicine, School of Medicine, 65672 Kyungpook National University, Daegu, Republic of Korea

**Keywords:** pleural infection, *Streptococcus anginosus* group, *Klebsiella pneumoniae*

## Abstract

**Objectives:**

Complicated pleural infections present significant challenges. Predominant causative microorganisms include the *Streptococcus anginosus* group (SAG) and *Klebsiella pneumoniae* (KP). However, limited data are available on the risk factors and outcomes associated with SAG-related pleural infection compared to KP-related pleural infection.

**Methods:**

This retrospective study was conducted in patients who underwent pleural drainage due to complicated pleural infection at Kyungpook National University Hospital in South Korea between January 2011 and December 2023. Clinical characteristics, drug resistance profiles, and outcomes were compared between patients with SAG-related and KP-related pleural infections.

**Results:**

A total of 432 patients were assessed. Among them, 161 (37 %) had positive pleural fluid cultures, with SAG (n=68, 42 %) and KP (n=34, 21 %) being the predominant pathogens. Thus, 102 patients with complicated pleural infection caused by SAG or KP were analyzed. SAG cases were associated with higher rates of chronic neurologic disease, lower rates of diabetes mellitus, prolonged symptom duration, elevated white blood cell counts, and positive gram stains on pleural fluid compared to KP cases. There were no significant differences observed between the two groups regarding radiological findings. SAG strains showed resistance rates exceeding 20 % to penicillin, erythromycin, tetracycline, and clindamycin, while remaining largely susceptible to commonly used third-generation cephalosporins, ampicillin, and fluoroquinolones. The in-hospital mortality rates were approximately 10 %, consistent across both groups.

**Conclusions:**

SAG-related pleural infections showed distinct clinical features, including more frequent chronic neurologic disease, but in-hospital mortality was comparable to that of KP-related infections.

## Introduction

Complicated pleural infections often develop as a complication of pneumonia, although they can also stem from hematogenous spread or the trans-diaphragmatic extension originating from intra-abdominal infection [1]. It is a significant condition with a relatively prolonged hospital stay and substantial mortality, requiring pleural drainage along with appropriate antibiotics [2]. Identifying the causative microorganism facilitates the use of suitable antibiotics and contributes to a favorable patient outcome. Nucleic acid sequencing utilizing 16S ribosomal ribonucleic acid has significantly improved the identification of causative microorganisms, whereas conventional culture methods typically confirm the pathogen in only 20–60 % of pleural fluid samples in cases of complicated pleural infection [3], [4], [5], [6], [7].

The prevalent causative microorganisms in pleural infections include anaerobic bacteria, the *Streptococcus anginosus* group (SAG), *Enterobacteriaceae, Staphylococcus aureus*, and others [5], [6], [7], [8], [9]. Anaerobic bacteria primarily constitute the pathogenic agents, especially in polymicrobial infections, and are seldom identified through conventional culture testing [5]. The SAG encompasses a subgroup of viridans *streptococci* including *S. anginosus*, *Streptococcus constellatus*, and *Streptococcus intermedius,* known for its high frequency of complicated purulent infections such as parapneumonic effusion or empyema [1], 7], 10], 11].

*Klebsiella pneumoniae* (KP), a representative organism among *Enterobacteriaceae*, is also recognized for its propensity to cause abscesses in various organs and is a frequent culprit in complicated pleural infections, particularly in Asia [1], [5], [6], [7], [8], [9]. Well-known risk factors associated with pleural infection caused by KP include diabetes mellitus (DM), liver cirrhosis, and lung cancer [12]. However, risk factors for SAG-associated pleural infection are not yet well-established. Additionally, little is known about the drug resistance profiles of SAG bacteria, which may potentially influence the initial choice of empirical antibiotics. Therefore, we conducted a comparison of clinical characteristics, drug resistance profiles, and outcomes between patients with complicated pleural infection caused by SAG and KP.

## Methods

### Study population

Consecutive patients who underwent pleural drainage for complicated parapneumonic effusion or empyema at Kyungpook National University Hospital, a tertiary referral hospital in South Korea, between January 2011 and December 2023 were retrospectively assessed. A complicated parapneumonic effusion was defined by a pleural fluid pH of less than 7.2, glucose less than 40 mg/dL, lactate dehydrogenase (LDH) greater than 1,000 U/L, or a positive Gram stain or culture, while empyema was defined by the presence of frank pus [2]. From this cohort, patients with positive pleural fluid cultures were further reviewed and those with monomicrobial infections due to either SAG or KP were included in the present study. Cases involving polymicrobial organisms, culture-negative samples, or other pathogens were excluded from the analysis. The study protocols were approved by the Institutional Review Board of Kyungpook National University Hospital.

### Data collection

Demographic information, comorbidities, infection acquisition site (community vs. hospital), blood and pleural fluid test results, causative pathogens and its drug susceptibility testing data, and in-hospital mortality details were extracted from the electronic medical records of patients with pleural infections caused by either SAG or KP. Comorbidities comprised malignancy, DM, advanced liver disease, chronic lung disease, and chronic neurologic disease. Chronic lung disease encompassed chronic obstructive pulmonary disease, bronchiectasis, and interstitial lung disease. Chronic neurologic disease was defined as the presence of epilepsy, Parkinson’s disease, Alzheimer’s disease, or cerebrovascular disease. The RAPID score (Renal, Age, fluid Purulence, Infection source, and Dietary) was also computed [13]. A thoracic radiologist and a pulmonary specialist retrospectively evaluated radiological findings, including the assessment of pleural effusion extent, loculation status on chest radiographs, and the presence of consolidation and cavitary lesions on chest computed tomography scans. A comparative analysis of clinical, laboratory, and radiological data, as well as outcomes, was performed between the SAG and KP groups.

### Statistical analysis

Statistical analyses were conducted using IBM SPSS Statistics Version 22.0 for Windows (IPM Corp., Armonk, NY, USA). Continuous variables were described as median and interquartile range (IQR) and compared using either the t-test or the Mann-Whitney U test, while categorical variables, presented as number and percentage, were compared using the chi-square or Fisher’s exact test. Univariable and multivariable logistic regression analyses were performed to identify factors associated with SAG bacteria in patients with complicated pleural infection caused by either SAG or KP. Odds ratio (OR) and 95 % confidence intervals (CI) were reported.

## Results

During the study period, 432 patients with complicated pleural infections who received pleural drainage were found. Among them, 161 (37 %) had a positive pleural fluid culture. The causative pathogens from the pleural fluid culture included as SAG (n=68, 42 %), KP (n=34, 21 %), *Staphylococcus* species (n=16, 10 %), non-SAG *Streptococcus* species (n=12, 7 %), anaerobic bacteria (n=11, 7 %), *Escherichia coli* (n=7, 4 %), *Enterococcus* species (n=5, 3 %), and others (n=12, 7 %). Polymicrobial infection was observed in 4 patients (2 %). Among SAG bacteria, *S. intermedius* was the most prevalent (n=39), followed by *S. constellatus* (n=24) and *S. anginosus* (n=5). In total, 102 patients with complicated pleural infection caused by SAG or KP were included in this study. Median age was 69 years and approximately 80 % of the patients were male (Table 1). In total, 33 % had comorbid DM, which was observed to be more prevalent in KP-related pleural infection cases than in SAG-related pleural infection cases (56 vs. 22 %, p=0.001). On the contrary, chronic neurologic disease was significantly frequent in cases with SAG-related pleural infection compared to those with KP-related pleural infection (32 vs. 12 %, p=0.025). Additionally, the frequency of symptom duration >2weeks was also frequent in SAG cases (p=0.030). There was no significant difference in the infection acquisition site between the two groups.

**Table 1:** Clinical, laboratory, and radiologic characteristics of patients with complicated pleural infection caused by *Streptococcus anginosus* group and *Klebsiella pneumoniae*.

Variable	Total (n=102)	*Streptococcus anginosus* group (n=68)	*Klebsiella pneumoniae* (n=34)	p-Value
Age, years	69 (59–78)	69 (58–79)	68 (62–77)	0.876
Male	80 (78)	55 (81)	25 (74)	0.395
Ever-smoker	62 (61)	41 (60)	21 (62)	0.886
Underlying diseases	74 (73)	48 (71)	26 (76)	0.530
Malignancy	7 (7)	5 (7)	2 (6)	1.0
Diabetes mellitus	34 (33)	15 (22)	19 (56)	0.001
Advanced liver disease	7 (7)	3 (4)	4 (11)	0.232
Chronic lung disease	15 (15)	12 (18)	3 (9)	0.236
Chronic neurologic disease	26 (25)	22 (32)	4 (12)	0.025
Symptom duration >2wk	29 (28)	24 (35)	5 (15)	0.030
Chest pain	70 (69)	46 (68)	24 (71)	0.763
Fever	73 (72)	50 (74)	23 (68)	0.535
Community acquired infection	89 (87)	60 (88)	29 (85)	0.877
Blood				
WBC count, cells/μL	17,330 (11,790–25,067)	18,705 (13,033–26,585)	12,440 (9,337–17,762)	0.002
CRP, mg/dL	20.2 (15.6–30.7)	21.6 (16.8–32.7)	19.9 (13.9–28.1)	0.149
BUN, mg/dL	21.4 (13.9–31.9)	20.5 (14.0–32.4)	23.2 (12.8–32.7)	0.739
Albumin, g/dL	2.7 (2.4–3.1)	2.7 (2.4–3.1)	2.6 (2.3–3.5)	0.704
LDH, U/L	222 (163–290)	219 (156–287)	229 (181–297)	0.396
RAPID score	4 (3–5)	3 (2–5)	4 (3–5)	0.088
Pleural fluid analysis				
WBC count, cells/μL	22,200 (3,570–79,494)	26,032 (4,614–95,345)	12,149 (1,881–50,884)	0.171
pH	6.76 (6.55–7.06)	6.75 (6.50–6.91)	6.92 (6.65–7.17)	0.026
Protein, g/dL	4.1 (2.8–5.0)	3.9 (2.2–4.9)	4.2 (3.6–5.1)	0.193
Glucose, mg/dL	5 (1–73)	2 (1–28)	80 (2–162)	<0.001
LDH, U/L	2,397 (1,150–7,772)	3,088 (1,330–9,436)	1,473 (826–4,543)	0.054
ADA, U/L	79 (45–206)	91 (48–230)	58 (38–128)	0.058
Positive gram stain	41 (40)	35 (51)	6 (18)	0.001
Positive blood culture	5 (5)	2 (3)	3 (9)	0.330
Empyema	44 (43)	36 (53)	8 (24)	0.005
Radiological findings				
Consolidation	64 (63)	42 (62)	22 (65)	0.772
Cavitary lesion	10 (10)	5 (7)	5 (15)	0.295
Large amount effusion^a^	31 (30)	22 (32)	9 (27)	0.543
Loculated effusion	87 (85)	60 (88)	27 (79)	0.236

Data are expressed as the median (interquartile range) or number (%). WBC, white blood cells; CRP, C-reactive protein; BUN, blood urea nitrogen; LDH, lactate dehydrogenase; RAPID, renal/age/purulent/infection source/dietary; ADA, adenosine deaminase. ^a^Was defined as effusion of more than two thirds of one hemithorax.

Patients with SAG-related pleural infection had significantly higher levels of white blood cell (WBC) count compared with those with KP-related pleural infection (median [IQR]: 18,705 [13,033–26,585] vs. 12,440 [9,337–17,762] cells/µL, p=0.002). The RAPID score tended to be higher in patients with KP-related pleural infection than in those with SAG-related pleural infection (median [IQR]: 4 [3], [4], [5] vs. 3 [2], [3], [4], [5], p=0.088).

The analysis of pleural fluid revealed that patients with SAG-related pleural infection had significantly reduced levels of pH and glucose compared to those with KP-related pleural infection. Similarly, the levels of LDH and adenosine deaminase tended to be higher in SAG-related pleural infection. In addition, patients with SAG-related pleural infection had a significantly higher rate of positive pleural fluid Gram stain results (51 vs. 18 %, p=0.001) and empyema (53 vs. 24 %, p=0.005), with a two to three-fold increase in occurrence compared to those with KP-related pleural infection. The rate of positive blood culture was 5 % in the total study population, with no significant difference between the two groups. Regarding radiological evaluation, including consolidation, cavitary lesions, the amount of pleural effusion, and loculation, there were no significant differences between the two groups.

Multivariable analysis revealed that chronic neurologic disease (OR=8.64 [95 % CI=1.26–59.37], p=0.028), symptom duration >2 weeks (OR=13.35 [95 % CI=1.90–93.89], p=0.009), blood WBC count (OR=9.02 [95 % CI=2.13–38.25], p=0.003), and positive pleural fluid Gram stain (OR=5.97 [95 % CI=1.40–25.51], p=0.016) were associated with an increased risk of SAG-related pleural infection (Table 2). Conversely, DM (OR=0.20 [95 % CI=0.05–0.81], p=0.024) and higher RAPID score (OR=0.46 [95 % CI=0.26–0.82], p=0.008) were associated with a decreased risk of SAG-related pleural infection.

**Table 2:** Multivariable analyses for factors associated with complicated pleural infection due to *Streptococcus anginosus* group.

	Multivariate analysis	
Variable	OR (95 % CI)	p-Value
Diabetes mellitus	0.20 (0.05–0.81)	0.024
Chronic neurologic disease	8.64 (1.26–59.37)	0.028
Symptom duration >2wk	13.35 (1.90–93.89)	0.009
Blood WBC (Ln)	9.02 (2.13–38.25)	0.003
Pleural fluid Gram stain (+)	5.97 (1.40–25.51)	0.016
RAPID score	0.46 (0.26–0.82)	0.008

OR, odds ratio; CI, confidence interval; WBC, white blood cells.

**Figure 1:**
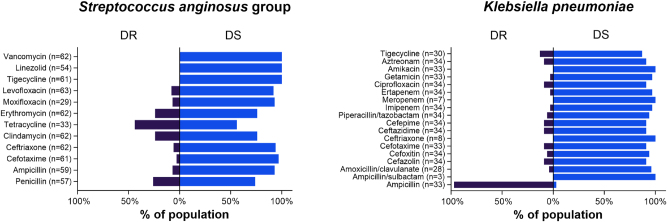
Antimicrobial susceptibility testing results in complicated pleural infection cases caused by *Streptococcus anginosus* group and *Klebsiella pneumoniae*. DR=drug resistant; DS=drug susceptible.

Resistance profiling to the tested antibiotics was depicted in cases of SAG and KP pleural infections (Figure 1). For SAG-related pleural infection cases, the rates of resistance to tetracycline, penicillin, clindamycin, and erythromycin each exceeded 20 %. Conversely, resistance to ampicillin, cefotaxime, ceftriaxone, moxifloxacin, and levofloxacin ranged from 3 to 8 %. In cases of KP-related pleural infection, ampicillin resistance reached 97 %, indicating an intrinsic resistance to ampicillin. The proportion of KP strains resistant to ampicillin/clavulanate, cefotaxime, imipenem, or ertapenem ranged from 3 to 8 %.

The predominantly utilized initial antibiotic regimen was ampicillin/sulbactam ± macrolides or fluoroquinolones (59 %), followed by piperacillin/tazobactam ± macrolides or fluoroquinolones (25 %) and cefotaxime or ceftriaxone ± macrolides or clindamycin (8 %) (Table 3). According to antibiotic susceptibility testing, the percentage of inappropriate initial antibiotic regimens was 5 %, and there was no discernible difference between the SAG and KP pleural infection cases. Around 40 % of patients underwent intrapleural fibrinolytic therapy, and this percentage was comparable between patients with SAG-related and KP-related pleural infection. The duration of pleural catheter drainage was also similar in both groups, with a median of 9 days (IQR, 6–14 days). Only a few patients underwent decortication surgery. The rates of in-hospital mortality were comparable between the two groups, standing at around 10 %. Following adjustment for the RAPID score, neither SAG nor KP was associated with an increased risk of in-hospital mortality.

**Table 3:** Initially chosen antibiotic regimens and clinical outcomes of patients with complicated pleural infection caused by *Streptococcus anginosus* group and *Klebsiella pneumoniae*.

Variable	Total (n=102)	*Streptococcus anginosus* group (n=68)	*Klebsiella pneumoniae* (n=34)	p-Value
Initial antibiotic regimens				0.168
Ampicillin/sulbactam ± macrolides or fluoroquinolones	60 (59)	45 (66)	15 (44)	
Piperacillin/tazobactam ± macrolides or fluoroquinolones	25 (25)	12 (18)	13 (38)	
Cefotaxime or ceftriaxone ± macrolides or clindamycin	8 (8)	5 (7)	3 (9)	
Meropenem ± vancomycin or fluoroquinolones	6 (6)	4 (6)	2 (6)	
Others	3 (3)	2 (3)^a^	1 (3)^b^	
Initial inappropriate antibiotics	5 (5)	2 (3)	3 (9)	0.330
Intrapleural fibrinolytic therapy	39 (38)	27 (40)	12 (35)	0.666
Time to PCD removal, d	9 (6–14)	10 (6–17)	8 (5–12)	0.294
Decortication operation	2 (2)	2 (3)	0 (0)	0.551
In-hospital mortality	11 (11)	7 (10)	4 (12)	1.0

Data are expressed as the median (interquartile range) or number (%). PCD, percutaneous catheter drainage. ^a^Cefepime (n=1) & moxifloxacin (n=1); ^b^Cefepime (n=1).

## Discussion

This study compared pleural infections caused by SAG and KP. SAG infections showed more intense inflammatory features in pleural fluid and were associated with chronic neurologic disease and longer symptom duration. However, in-hospital mortality was similar between the two groups, and microbial etiology was not independently associated with short-term outcomes. Given the limited data from previous small case series [8], 10], 11], our findings offer new insights into the clinical presentation and outcomes of SAG-related pleural infection.

DM has previously been shown to be associated with KP pleura infection [12], which aligns with our findings. In contrast, chronic neurological diseases were independently associated with SAG-related pleural infection in this study. In individuals with chronic neurological conditions prone to micro-aspiration, SAG, a normal commensal in the oral cavity or gastrointestinal tract, may contribute to the development of pneumonia followed by pleural infection [1], 10], 14]. However, a recent study suggested that the route of infection for bacteria causing oral-type pleural infections, including *S. intermedius* and anaerobic bacteria, is likely hematogenous spread from odontogenic infections rather than aspiration [15]. Although dental evaluation data were unavailable in this study, the frequency of positive blood culture testing or radiologic findings including consolidation and cavitary lesion were comparable between cases of SAG-related and KP-related pleural infections. These findings support the hypothesis that SAG-related pleural infections are more likely to result from aspiration rather than hematogenous spread [16], 17]. Further investigation is deemed necessary to explore the infection route of SAG pathogens in pleural infection patients with chronic neurologic disease.

Patients with SAG-related pleural infection exhibited prolonged symptom duration before admission, aligning with the consistent finding of a higher proportion of empyema cases and chronic neurologic disease. Likewise, these patients presented with a higher WBC count in blood and lower pH and glucose levels in pleural fluid. Furthermore, the frequency of a positive Gram stain in pleural fluid was significantly higher in patients with SAG-related pleural infection. These observations may assist clinicians in predicting the causative pathogen and making decisions regarding empirical antibiotic treatments.

The treatment of choice for SAG-related pleural infection has not yet been established, SAG bacteria seem to be generally susceptible to β-lactam agents [18]. The initial antibiotics selected in this study aimed to account for the potential of coinfection with anaerobic bacteria. Most of these antibiotics proved effective against the isolated pathogens in both SAG and KP groups. In the SAG group, one *S. intermedius* and one *S. constellatus* exhibited concurrent resistance to ampicillin, erythromycin, cefotaxime, and ceftriaxone. These cases, identified as community-acquired infections, were treated with a modified regimen including vancomycin or meropenem, and recovered without complications. In the KP-related pleural infection group, three strains were identified as extended-spectrum beta-lactamase producing bacteria. These patients also showed favorable clinical outcomes after modifying their antibiotic treatment.

Only 2 % of patients in our cohort underwent surgical management beyond pleural drainage and antibiotic therapy. The low rate of surgical intervention likely reflects a combination of clinical and institutional factors, including the advanced age and comorbidity burden of the patients, a predominantly conservative treatment strategy at our center, and the increased effectiveness of advanced catheter-based drainage techniques, such as multiple catheter insertions and intrapleural fibrinolytic therapy [7], 19]. Notably, the observed in-hospital mortality rate of 11 %, despite a median RAPID score of 4 (corresponding to an expected 3-month mortality of ∼18 %), suggests that this conservative approach did not adversely affect clinical outcomes [20].

While previous studies, including those from the TORPIDS cohort [5] and Wong et al. [7], reported improved long-term survival in patients with SAG-related pleural infections, our study did not observe a significant difference in in-hospital mortality between the SAG and KP groups. This discrepancy may be attributed to several factors, including the higher baseline severity of illness in our cohort, differences in comparator groups (KP vs. non-SAG organisms), and the limited statistical power of our analysis. Moreover, differences in mortality related to microbial virulence may become more apparent over longer-term follow-up, which was beyond the scope of our study focused on in-hospital outcomes.

The limitations of this study include its retrospective design and single-center setting, which may introduce selection bias. In addition, the detection rate of anaerobic pathogens and mixed infections was relatively low, likely due to reliance on conventional culture methods. Furthermore, the analysis was limited to patients with monomicrobial pleural infections caused by either SAG or KP, and therefore may not be generalizable to culture-negative or non-SAG/KP infections. However, this study contributes one of the more substantial datasets on SAG-related pleural infection currently available in the literature.

In summary, patients with SAG-related pleural infection were more likely to have chronic neurologic disease, prolonged symptom duration, and positive Gram stain results in pleural fluid. These characteristics, together with elevated systemic and pleural inflammatory markers, support an aspiration-related pathogenesis rather than hematogenous spread. Despite these distinct clinical features, the in-hospital mortality rate was comparable to that observed in patients with KP-related pleural infection.
